# Genetic variants linked to the phenotypic outcome of invasive disease and carriage of *Neisseria meningitidis*

**DOI:** 10.1099/mgen.0.001124

**Published:** 2023-10-24

**Authors:** Lorraine Eriksson, Thor Bech Johannesen, Bianca Stenmark, Susanne Jacobsson, Olof Säll, Sara Thulin Hedberg, Hans Fredlund, Marc Stegger, Paula Mölling

**Affiliations:** ^1^​ Department of Laboratory Medicine, Faculty of Medicine and Health, Örebro University, Örebro, Sweden; ^2^​ Department of Bacteria, Parasites and Fungi, Statens Serum Institut, Copenhagen, Denmark; ^3^​ Department of Infectious Diseases, Faculty of Medicine and Health, Örebro University, Örebro, Sweden

**Keywords:** Carriage, Genome-wide association study, Invasive meningococcal disease, *Neisseria meningitidis*

## Abstract

*
Neisseria meningitidis* can be a human commensal in the upper respiratory tract but is also capable of causing invasive diseases such as meningococcal meningitis and septicaemia. No specific genetic markers have been detected to distinguish carriage from disease isolates. The aim here was to find genetic traits that could be linked to phenotypic outcomes associated with carriage versus invasive *N. meningitidis* disease through a bacterial genome-wide association study (GWAS). In this study, invasive *N. meningitidis* isolates collected in Sweden (*n*=103) and carriage isolates collected at Örebro University, Sweden (*n*=213) 2018–2019 were analysed. The GWAS analysis, treeWAS*,* was applied to single-nucleotide polymorphisms (SNPs), genes and k-mers. One gene and one non-synonymous SNP were associated with invasive disease and seven genes and one non-synonymous SNP were associated with carriage isolates. The gene associated with invasive disease encodes a phage transposase (NEIS1048), and the associated invasive SNP *glmU* S373C encodes the enzyme N-acetylglucosamine 1-phosphate (GlcNAC 1-P) uridyltransferase. Of the genes associated with carriage isolates, a gene variant of *porB* encoding PorB class 3, the genes *pilE*/*pilS* and *tspB* have known functions. The SNP associated with carriage was *fkbp* D33N, encoding a FK506-binding protein (FKBP). K-mers from *PilS*, *tbpB* and *tspB* were found to be associated with carriage, while k-mers from *mtrD* and *tbpA* were associated with invasiveness. In the genes *fkbp*, *glmU*, *PilC* and *pilE*, k-mers were found that were associated with both carriage and invasive isolates, indicating that specific variations within these genes could play a role in invasiveness. The data presented here highlight genetic traits that are significantly associated with invasive or carriage *N. meningitidis* across the species population. These traits could prove essential to our understanding of the pathogenicity of *N. meningitidis* and could help to identify future vaccine targets.

## Data Summary

The authors confirm all supporting data, codes and protocols have been provided within the article. Illumina sequencing data have been uploaded to the European Nucleotide Archive (ENA) EMBL-EBI under accession number PRJEB61085 (https://www.ebi.ac.uk/ena/browser/view/PRJEB61085). Genetic typing of the included isolates was performed using the PubMLST database (https://pubmlst.org/) and PubMLST IDs are available in Table S1, in the online version of this article.

**Impact Statement**

*
Neisseria meningitidis* is a bacterium that can be commensal and can also cause severe disease in humans. Currently no identified target allows genetics-based determination of an isolate’s ability to cause invasive disease. In this study, we aimed to identify genetic markers that could distinguish between invasive and carriage isolates. Such markers could be used to determine the potential of an isolate to cause severe disease and to identify potential vaccine targets aimed at more virulent *N. meningitidis* variants.

## Introduction

*Neisseria meningitidis* is a human commensal that colonizes the upper respiratory tract and can cause invasive meningococcal disease (IMD), usually presenting as septicaemia or meningitis [1]. Twelve different polysaccharide capsules can be expressed by *N. meningitidis *, and these can be switched between isolates, but can also undergo phase variation [2–7]. The polysaccharide capsule is used to classify the bacteria into serogroups, and IMD is usually caused by isolates in the serogroups A, B, C, W, X and Y [2]. It is uncommon for invasive isolates to not express a capsule, but carriage isolates can be capsulated, non-capsulated (cnl) and non-groupable (NG) [2, 8]. *N. meningitidis* can further be typed by multilocus sequence typing (MLST) to assign isolates to sequence type (ST) and clonal complex (CC) [9], which is now often done using whole-genome sequencing (WGS), which also allows detailed investigation of putative outbreaks and genetic traits in general [10, 11].

The distribution of serogroups causing IMD varies around the world. In many countries, serogroups B and C predominate, but serogroups W and Y are also prevalent in some regions [12]. The serogroups W and Y currently cause most of the IMD in Sweden. In 2018, serogroup W represented 47 % (22/47) and serogroup Y 32 % (15/47) of all IMD cases in Sweden [13]; in 2019, 39 % (22/57) and 35 % (20/57) of IMD cases were caused by serogroups W and Y, respectively [14]. Carriage of *N. meningitidis* is estimated to be ~10 % in non-endemic areas [2] and is highest among adolescents [15, 16]. In Sweden, the carriage rate was recently shown to be 9 % in a cohort of university students at Örebro University, with a median age of 23 years [17]. During carriage, *N. meningitidis* colonizes the mucosal epithelium of the upper respiratory tract and occasionally invades the bloodstream and causes IMD. The underlying factors that drive this transition from commensal to invasive is still unclear; however, it is probably facilitated by the virulence of the bacteria and host genetic factors [2, 18, 19]. Virulence factors suggested to be important in invasiveness include the polysaccharide capsule [2, 3], and some isolates in so-called hypervirulent lineages (e.g. CC11) have been suggested to be more virulent than others [2]. Most genes present in *N. meningitidis* are shared between pathogenic and non-pathogenic *Neisseria *, and so far no distinct genes have been identified that can predict whether an isolate will be invasive [20]. In the genome-wide association study (GWAS) approach, genetic variants are investigated to identify their possible associations with specific traits of interest, such as phenotypes or clinical outcomes [21, 22]. Bacterial GWASs are affected by the population structure and the homologous recombination that occur in bacteria, but tools are available to adjust for these [21, 23].

The aim of this study was to compare contemporary *N. meningitidis* carriage and invasive isolates genetically using a GWAS approach to identify genetic variants linked to the phenotypic outcome of disease or carriage.

## Methods

### *N. meningitidis* isolates

In total, 316 *N *. *meningitidis* isolates were included in this study. The isolates comprised all invasive Swedish isolates from 2018 and 2019 (*n*=103), as well as carriage isolates (*n*=213) collected during a carriage study conducted at Örebro University in Sweden during September 2018 to September 2019 [17]. The mean age of the invasive isolates was 47 years (range 0–94 years) and the mean age of the carriage isolates was 24 years (range 19–38 years). Isolates had been WGS as part of routine diagnostics or during the carriage study on the Illumina platform, as described previously [17, 24]. STs, CCs and genogroups for all samples were retrieved from the *Neisseria* PubMLST database (www.pubmlst.org); information on all isolates is available in Table S1.

### GWAS

Prior to analysis, the sequencing data were quality controlled using bifrost (github.com/ssi-dk/bifrost) and contamination was checked with Kraken v 1.0 [25]. Single-nucleotide polymorphisms (SNPs) were identified in the core genome using NASP v1.0.0 [26] by aligning the raw sequencing data to the closed *N. meningitidis* NM3686 reference genome (GenBank accession ID CP009418.1). Only high-quality SNPs were retained by exclusion of any site with a minimum coverage of <10 or <90 % presence of the base calls of individual isolates. A phylogenetic tree was constructed using RAxML [27] based on the detected SNPs, pruned for recombination using Gubbins v 2.3.4 [28], and visualized using iTOL v 4.3 [29]. For gene presence/absence in relation to phenotype, the genomes were assembled using SPAdes v 3.11.1 [30], annotated with Prokka v 1.12 [31], using default settings, and accessory and core genomes were identified with Roary v 3.11.2 [32]. Genes were categorized as identical if they exhibited ≥95 % sequence similarity. The presence/absence of suggested virulence genes [33, 34] was also investigated in the GWAS analysis. These genes were searched for in the SPAdes assemblies by BLASTN searches in Biomatters Geneious Prime v 2019.1 (Biomatter Ltd, Auckland, New Zealand), using >90 % sequence similarity and coverage. For the k-mer approach, all k-mers with a length of 31 nucleotides were counted using fsm-lite v 1.0 (https://github.com/nvalimak/fsm-lite), and each unique pattern of presence/absence was tested using a GWAS approach. These results have been multiply test-corrected according to the unique patterns of presence/absence.

**Fig. 1.**
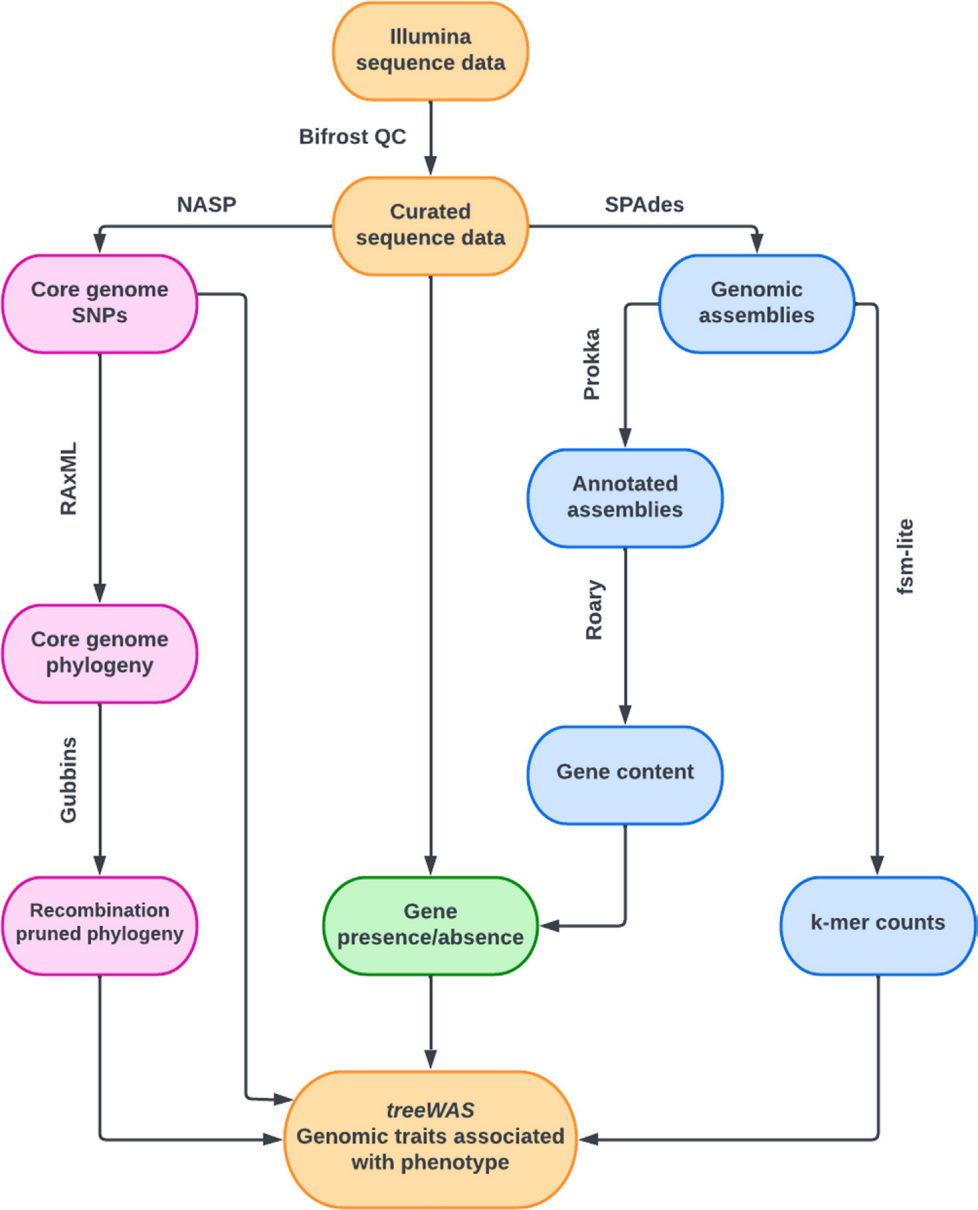
Overview of the bioinformatics workflow. The Illumina-generated reads were quality checked and used to obtain data on single-nucleotide polymorphisms (SNPs) in the core genome, k-mers and the absence or presence of genes.

TreeWAS V 1.0 was applied using R V 3.5.1 (www.r-project.org), and all used scripts are available in github (https://github.com/caitiecollins/treewas) [23]. To identify genomic traits associated with invasive or carriage phenotypes, we applied a GWAS approach using treeWAS to SNPs in the core genome, presence/absence data on accessory genes and, to also account for minor genetic variation within accessory genes, presence/absence of k-mers found across all samples. Significant genetic variants were identified with treeWAS based on a *P* threshold of 0.05 after Bonferroni adjustment for multiple testing. Figure 1 displays an overview of the bioinformatics workflow performed in this study. Key genomic traits were visualized on phylogenies using iTOL v 4.3 [29]. TreeWAS evaluates genotype–phenotype association for each locus using three different scores: the terminal score, which examines genotype–phenotype correlation with no regard to phylogentetic reconstruction; the simultaneous score, which infers ancestral states at each branch in the phylogeny and examines how often a change in genotype happens in parallel with a change in phenotype; and the subsequent score, which examines the proportion of tree branches where the genotype and phenotype coexist according to the ancestral state reconstruction. To measure the statistical significance of the score at each locus, a randomly simulated dataset with the same clonality, terminal phenotype and homoplasy distribution as the real dataset is generated, and each locus is scored to obtain a null distribution of scores in a dataset with no true associations to phenotype. The scores from the real dataset are then assigned *P*-values according to how they fit in the null distribution.

Significant genes and SNPs identified with the GWAS analysis were queried in the *Neisseria* typing database in PubMLST [35] to identify the corresponding NEIS genes.

### Identification of phage genes

The ORFs of the meningococcal disease-associated (MDA) phage [36, 37] (https://www.ncbi.nlm.nih.gov/nuccore/AL157959.1#) were identified by querying the sequences in PubMLST. Each MDA phage ORF corresponded to several NEIS genes; see Table S2. Each sample included in this study was searched in PubMLST for the presence of the NEIS genes corresponding to the MDA phage ORFs.

## Results

### Phylogeny of *N. meningitidis* invasive and carriage isolates

**Fig. 2.**
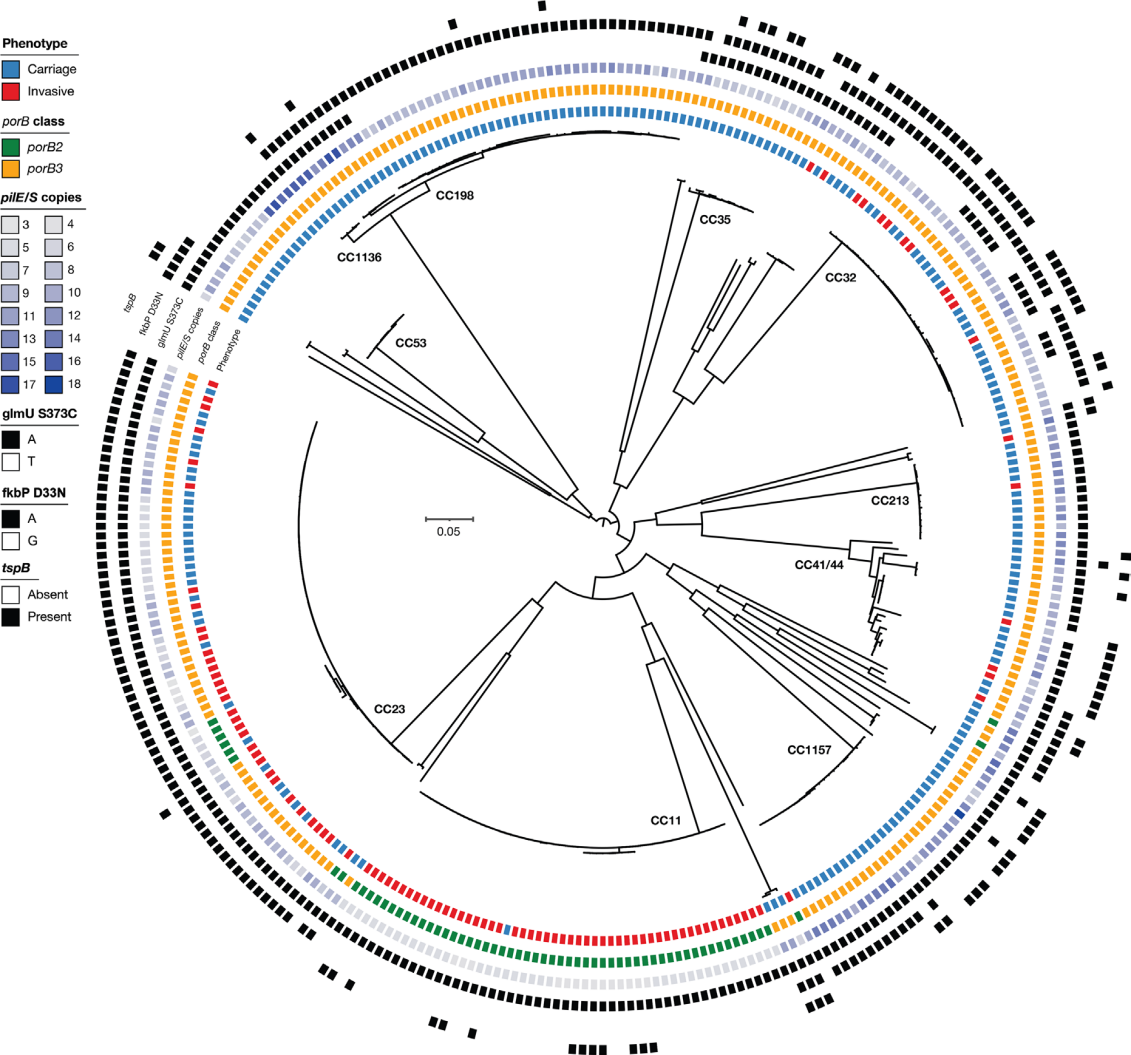
Phylogeny displaying the distribution of associated single-nucleotide polymorphisms (SNPs) and genes in treeWAS. Rings display the phenotype, presence of *porB* class, copies of *pilE/S*, and presence of *glmU* S373C, *fkbp* D33N and *tspB*. Clonal complexes (CCs) are presented on the corresponding branches of the tree. The phylogeny is based on SNPs in the core genome covering 68 % of the reference.

**Fig. 3.**
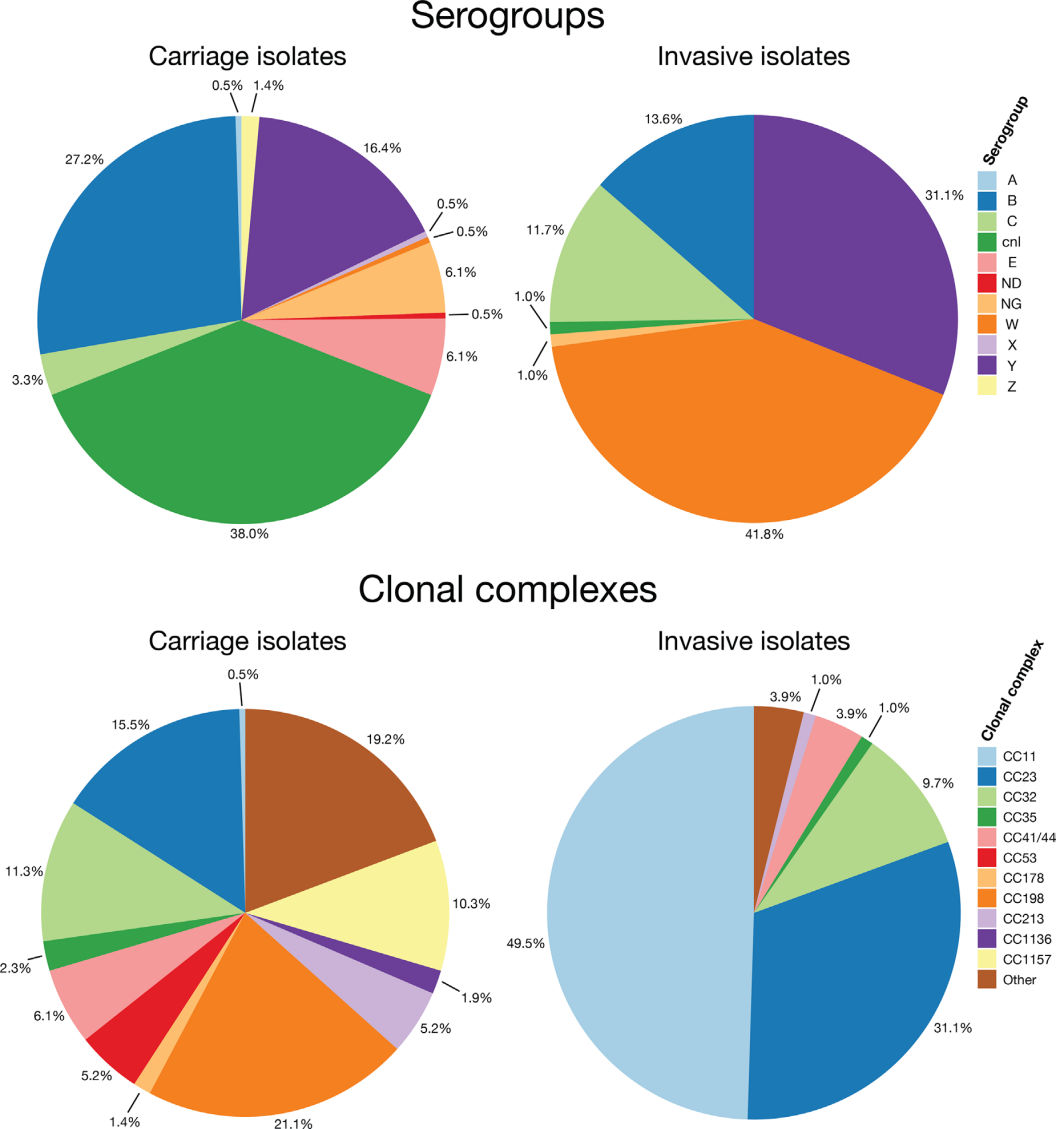
Pie charts display the distribution and percentage of clonal complexes (CCs) and serogroups among invasive and carriage isolates.

The phylogeny of invasive (*n*=103) and carriage *N. meningitidis* isolates (*n*=213) is displayed in Fig. 2, with information on phenotype and CC. Pie charts in Fig. 3 show the distribution of serogroups and CCs between invasive and carriage isolates. The invasive isolates belonged to serogroup W (*n*=43, 42 %), serogroup Y (*n*=32, 31 %), serogroup B (*n*=14, 14 %) and serogroup C (*n*=12, 12 %); the major CCs were CC11 (*n*=51, 50 %), CC23 (*n*=32, 3 %) and CC32 (*n*=10, 10 %). The carriage isolates belong to different serogroups; the most common were B (*n*=58, 27 %), Y (*n*=35, 16 %), E (*n*=13, 6 %) and C (*n*=7, 3 %). Several of the isolates did not express a capsule (cnl, *n*=81, 38 %). The isolates belonged to many different CCs, with the most common being CC198 (*n*=45, 21 %), CC23 (*n*=33, 16 %) and CC32 (*n*=24, 11 %). Complete information on the included isolates is available in Table S1.

### Genetic variants identified with GWAS

#### Significant SNPs

With treeWAS, 31 significant SNPs were identified in the simultaneous test, but none in the terminal and subsequent tests (Table 1, Fig. S1). Two SNPs, *fkbP* D33N and *glmU* S373C, were non-synonymous. The SNP *glmU* S373C was associated with invasive isolates (*P*<0.001). The gene *glmU* (NEIS0015) encodes the enzyme N-acetylglucosamine 1-phosphate (GlcNAC 1-P) uridyltransferase [38] (https://www.uniprot.org/uniprotkb/Q9K1P3/entry) that is involved in the synthesis of UDP-N-acetylglucosamine pyrophosphorylase (UDP-GlcNAc) [38]. In *N. meningitidis* and *Neisseria gonorrhoeae,* UDP-GlcNAc is a substrate involved in the synthesis of lipooligosaccharide (LOS) [39], polysaccharide capsule [40] and CMP-NANA, which is the substrate for sialic acids [41]. Sialic acids can be part of the polysaccharide capsule [42] and added to LOS [43], which may help the bacteria evade the immune system of the host by mimicry, masking surface antigens, and binding factor H to fragments of C3 on the bacterial surface [44, 45]. The availability of UDP-GlcNAc may affect the growth of the bacteria, since it is also involved in the synthesis of cell wall components [46].

**Table 1.** Single-nucleotide polymorphisms (SNPs) identified as associated with invasive or carriage isolates with the simultaneous test in treeWAS (all SNPs had *P*<0.001)

Gene	Product	Reference base	Alternative base	SNP position	Mutation	Association
NEIS0004 (fbp)*	Peptidyl-prolyl *cis*-*trans* isomerase	G	A	97	D33N	Carriage
NEIS0015 (glmU)*	UDP-N-acetylglucosamine pyrophosphorylase	A	T	1117	S373C	Invasive
NEIS0015 (glmU)	UDP-N-acetylglucosamine pyrophosphorylase	C	A	72	P24P	Invasive
NEIS0015 (glmU)	UDP-N-acetylglucosamine pyrophosphorylase	C	A	129	A43A	Invasive
NEIS0015 (glmU)	UDP-N-acetylglucosamine pyrophosphorylase	T	C	186	V62V	Invasive
NEIS0015 (glmU)	UDP-N-acetylglucosamine pyrophosphorylase	T	G	390	V130V	Carriage
NEIS0015 (glmU)	UDP-N-acetylglucosamine pyrophosphorylase	T	C	474	N158N	Invasive
NEIS0015 (glmU)	UDP-N-acetylglucosamine pyrophosphorylase	T	C	1104	D368D	Invasive
NEIS0038	Putative lipoprotein	T	A	358	K120K	Invasive
NEIS0038	Putative lipoprotein	A	T	354	C118C	Invasive
NEIS0038	Putative lipoprotein	A	T	351	D117D	Invasive
NEIS0038	Putative lipoprotein	A	T	345	G115G	Invasive
NEIS0038	Putative lipoprotein	T	A	341	E114E	Invasive
NEIS0168 (lpxA)	UDP-N-acetylglucosamine acyltransferase	G	A	618	E206E	Carriage
NEIS0251	NADH dehydrogenase subunit L	A	T	1227	Y409Y	Carriage
NEIS0251	NADH dehydrogenase subunit L	A	C	1006	L336L	Carriage
NEIS0375	Putative NADH : FMN oxidoreductase	G	T	57	C19C	Carriage
NEIS0326	Leucyl-tRNA synthetase	A	C	2496	T832T	Carriage
NEIS0541	Maf-like protein	A	G	534	G178G	Carriage
NEIS0774	Putative ATP-dependent protease ATP-binding protein	G	C	1518	V506V	Invasive
NEIS1355	Putative ATP-dependent RNA helicase	A	G	324	L108L	Carriage
NEIS1553 (lptA)	Lipid A phosphoethanolamine transferase	C	T	957	T319T	Invasive
NEIS1948	Chaperonin GroEL	C	T	96	G32G	Carriage
NEIS1948	Chaperonin GroEL	A	G	114	V38V	Carriage
NEIS1948	Chaperonin GroEL	C	G	117	V39V	Carriage
NEIS1948	Chaperonin GroEL	C	T	105	G53G	Invasive
NEIS1948	Chaperonin GroEL	A	G	255	A85A	Carriage
NEIS1948	Chaperonin GroEL	C	T	318	Y106Y	Invasive
NEIS1948	Chaperonin GroEL	C	A	327	A109A	Invasive
NEIS2030	Putative periplasmic protein	A	C	1453	R485R	Carriage
NEIS2030	Putative periplasmic protein	T	C	1602	S534S	Carriage

*Non-synonymous SNPs.

The SNP *fkbP* D33N was associated with carriage isolates (*P*<0.001). The *fkbP* gene (NEIS0004) encodes an enzyme that belongs to the FK506-binding protein (FKBP) family, which is part of the peptidyl-prolyl *cis*-*trans* isomerase (PPIase) protein superfamily [47]. These enzymes are involved in protein folding and have many important functions in cells; some PPIases have also been identified as virulence factors and as being of interest in designing drugs targeting virulence [48].

#### Significant genes

Significant genes were identified in all three tests performed with treeWAS (Fig. S2). A phage transposase (NEIS1048) was associated with invasive isolates, and the genes *porB* (NEIS2020), NEIS2411, NEIS1667, NEIS1668, *pilE/S*, *tspB* (NEIS0025, NEIS1715, NEIS1866) and NEIS0975 were associated with carriage isolates (Table 2).

**Table 2.** Genes associated with invasive and carriage isolates in the treeWAS. All genes had *P*<0.001

Gene identified by treeWAS	NEIS gene (PubMLST)	Product (PubMLST)	TreeWAS test	Association
*porB*	NEIS2020	PorB, porin, major outer membrane protein (class 3)	Terminal	Carriage
group_428	NEIS2411	Putative DNA-binding protein	Terminal	Carriage
group_2772	NEIS1667	Hypothetical protein	Simultaneous	Carriage
group_954	NEIS1668* allele 4	Putative cell surface protein (truncated TspB)	Simultaneous	Carriage
*pilE/pilS*	NEIS0210† (allele 1045)	PilE	Simultaneous	Carriage
group_32	NEIS0025 NEIS1715 NEIS1866	TspB protein	Simultaneous	Carriage
*porB*	(NEIS2020)	PorB, porin, major outer membrane protein (class 3)	Subsequent	Carriage
group_2763	NEIS0975	Pseudo	Subsequent	Carriage
group_2041	NEIS1048	Phage transposase	Subsequent	Invasive

*No exact match, closest match was allele 4 of NEIS1668 with a three nuclotide difference in alignment.

†No exact match, closest match was allele 1045 of NEIS0210 with a 13 allele difference and 2 gaps in the alignment.

The gene *porB* (NEIS2020) was associated with carriage isolates. This gene encodes the outer membrane protein PorB, a porin that allows transportation through the membrane, but that has also been suggested to be involved in invasiveness [49, 50], antibiotic resistance [51], intracellular survival and apoptosis of host cells [52]. This protein exists in two variants: class 2 and class 3. All 316 isolates (carriage and invasive) had 1 copy of either *porB* encoding PorB class 2 (PorB2) or class 3 (PorB3) (Fig. 2). The *porB* variant encoding class 3 was associated with carriage isolates by both the terminal and subsequent scores (*P*<0.001 after multiple testing correction for both). Conversely, the *porB* class 2 variant had high association with invasive isolates, but not high enough to meet the threshold for significance after multiple testing correction. The result is nevertheless noteworthy, with non-adjusted *P*<0.001 for both terminal and subsequent scores.

The gene *tspB* was associated (*P*<0.001) with carriage isolates and encodes the TspB (T-cell- and B-cell-stimulating) protein. Three genes (NEIS0025, NEIS1715 and NEIS1866) all encode TspB [53, 54]. In treeWAS*, tspB* was identified as one gene that consists of a cluster of genes with different lengths that are variants of *tspB* with ≥95 % similarity. This gene was not present among most of the invasive serogroup W CC11 and serogroup Y CC23 isolates (Fig. 2). TspB is expressed on the surface of the bacteria, where it binds human IgG, and it has been observed to be involved in the survival of serogroup B *N. meningitidis* in human serum [53]. The *tspB* gene is part of the MDA phage that may exist in up to four copies in the meningococcus [36, 55]. To investigate whether the isolates included in this study had a complete MDA phage, the NEIS genes corresponding to the eight ORFs of MDA phage [37] were searched for in the isolates. Each ORF corresponded to several NEIS genes (Table S2), and isolates were considered to have a complete MDA phage if at least one gene corresponding to each ORF was present. Genes corresponding to the complete MDA phage ORFs were present in 11 (11 %) of the invasive and 27 (13 %) of the carriage isolates. The MDA phage has previously been associated with invasive CC (e.g. CC11, CC35, CC23, CC32, CC41/44), and more uncommon among CCs considered to be non-invasive (e.g. CC198, CC269, CC4821) [37]. Here, the MDA phage was slightly more present among invasive CCs (47 %, 18/38) (consisting of both invasive and carriage isolates) than non-invasive CCs (34 %, 13/38). Many isolates contained one or more of the MDA phage genes, but not a complete MDA phage. Some isolates also had genes that were incomplete according to WGS.

TreeWAS identified a *PilE* gene associated (*P*<0.001) with carriage isolates, but this was in fact a cluster of genes containing both class 1 *pilE* genes and highly similar *pilS* loci. The closest match was allele 1045 of NEIS0210. The *pilE* gene encodes PilE, the major pilin in the type IV pili in *Neisseria *, which is involved in adhesion to endothelial cells, aggregation and DNA exchange [56, 57]. The *pilS*, a silent pili gene, is used for antigenic variation of PilE and thereby variation of the type IV pili. PilE can be separated into class 1 or 2 by the structure of the protein [58]. The genetic structures of these classes are also different. Class 1 has the *pilE* gene adjacent to a *pilS* cassette consisting of several *pilS* genes; in class 2 the *pilE* and *pilS* genes are present in different regions of the genome [59]. *PilS* loci resembling class 1 *pilE* were found in higher copy numbers in carriage isolates than in invasive isolates, while class 2 *pilE* was found almost exclusively in CC11. While class 1 *pilE* were found to be associated with carriage, class 2 *pilE* had very high association scores with invasive isolates, though not enough to meet the significance threshold of *P*<0.05 after multiple testing correction.

#### k-mers

A total of 8 539 248 unique 31-mers were identified across the dataset, of which 4 151 217 had a prevalence of 5–95 %. These represented 264 487 unique patterns of presence/absence and were tested for association with invasiveness and carriage using treeWAS. The number of associated k-mers in different genes is displayed in Table 3. Some of the genes have k-mers associated with both carriage and invasive isolates; this may suggest that specific variation within these genes can play a role in invasiveness.

**Table 3.** Number of k-mers in genes associated with invasive and carriage isolates, identified with treeWAS. All k-mers had *P*<0.05 after after Bonferroni adjustment for multiple testing

Gene	No. of associated k-mers	Association
*pilE* class 1	15	Carriage
*pilE* class 2	12	Invasive
*pilS* cassette	29	Carriage
*mtrD*	4	Invasive
*tspB*	7	Carriage
*tbpA* (tbp1)	5	Invasive
*tbpB* (tbp2)	10	Carriage
*glmU*	1/1	Carriage/invasive
*fkbp*	3/3	Carriage/invasive
*pilC*	2/4	Carriage/invasive

In components of the type IV pilus system, the *pilC*, *pilE* and *pilS* genes, k-mers were found that were associated with both carriage and invasive isolates. The transferrin-binding protein A (*tbpA*) was found to be associated with invasive isolates, whilst the transferrin-binding protein B (*tbpB*) was found associated with carriage isolates [60]. A sequence of the *tspB* gene that corresponds to the IgG-binding domain of the TspB protein (NCBI refseq ID WP_002215798.1 [53]) was found among the carriage-associated k-mers. Associations found for k-mers also support the association of the SNP *fkbp* D33N with carriage isolates. Carriage-associated k-mers (*n*=3) contained the SNP *fkbp* D33N, while k-mers associated with invasive isolates (*n*=3) did not contain this SNP. The *mtrD* (NEIS1633) gene encoding an inner membrane protein in the multiple transferable resistance (Mtr) system, which is involved in resistance to antibiotics [61], was associated with invasive isolates. A single k-mer from *glmU* was found to be associated with carriage, while another single k-mer was associated with invasive isolates.

## Discussion

In this study, we compared *N. meningitidis* invasive and carriage isolates to identify genetic traits associated with invasiveness or carriage using treeWAS, a GWAS approach that adjusts for lineage effects and can be used to avoid identifying traits that are only associated with the major lineages.

Fewer associations were found among invasive isolates compared to carriage isolates in this study. The invasive isolates had low genetic diversity, which made the dataset small, and it was more difficult to pinpoint virulent properties among these invasive isolates. The carriage isolates were genetically more diverse, which made the dataset larger and made it easier to identify associations. Several genes and k-mers were identified as being associated with carriage isolates, including *porB*, *tspB*, *pilE/pilS* and *tbpB* (Tables 2 and 3). The invasive isolates were associated with a phage transposase gene and k-mers in the genes *pilE*, *mtrD* and *tbpA* (Tables 2 and 3). Carriage and invasive isolates were both associated with one non-synomous SNP; *fkbp* D33N and *glmU* S373C, respectively.

The gene *glmU* encodes an enzyme involved in UDP-GalNAc synthesis. UDP-GalNAc is involved in several surface structures on *N. meningitidis* [39–41]. One of these structures is sialic acids that *N. meningitidis* can utilize to evade the immune system through mimicry and binding of factor H, which downregulates the alternative complement pathway [42, 43]. However, the presence of sialic acids on LOS may also interfere with binding to epithelial cells [62–64]. The importance of factor H binding in evading immune responses is seen from the number of proteins that can bind factor H: the factor H-binding protein (Fhbp), NspA and PorB [65, 66]. A study by Earle *et al*. [67] found an association between factor H and invasive isolates in a bacterial GWAS performed on *N. meningitidis *. The importance of factor H in invasive disease was also observed in a GWAS conducted on patients with IMD [68]. It was suggested in an infant rat model that *N. meningitidis* expressing PorB class 3 were less virulent during infections, perhaps in part because this class of PorB is less efficient in binding factor H. In contrast, PorB class 2 was found to exhibit enhanced factor H binding, and these isolates were more virulent in infections in the same infant rat model [65, 69]. Here, we found that PorB class 3 was associated with carriage isolates, which may suggest that factor H binding is less important in carriage than in invasive disease. However, PorB has several additional functions as a virulence factor that may be beneficial for both invasive and carriage isolates.

The carriage-associated *tspB* gene encoding the protein TspB, a surface-exposed protein that binds human IgG, can also be used to evade the immune system [53]. This gene was uncommon among isolates from serogroups W CC11 and Y CC23. The association of this gene with carriage was also confirmed by k-mers corresponding to the IgG-binding domain of the TspB protein. Earle *et al*. also found SNPs in the IgG-binding domain of TspB that were associated with carriage derived k-mers [67]. The *tspB* is part of the MDA phage, which was initially discovered in isolates belonging to invasive CCs [53, 54, 67]. We observed that genes corresponding to the MDA phage ORF were slightly more common among invasive CCs than carriage CCs and present at almost the same percentage among invasive and carriage isolates. However, because many of the genes corresponding to the MDA phage were incomplete based on WGS data and excluded from comparison, the number of isolates with a complete phage may be higher for both carriage and invasive isolates. Recent studies have found the MDA phage among carriage isolates and other non-pathogenic *Neisseria* species [36] and it has also been suggested to be involved in colonization [70]. These results are more in line with our findings.

In this study, type IV pili genes (*pilE* and *pilS*) were identified as being associated with carriage isolates. PilE is the major protein of this pili, and *pilS* is used as a reservoir for *pilE* antigenic variation. Type IV pili genes are important for initial attachment to cells, aggregation and DNA exchange [56, 57]. The *pilS* loci were previously believed to lack promoter elements, but some of them seem to be transcriptionally active in *N. gonorrhoeae* [71]. We found that the carriage isolates had more copies of *pilE*/*pilS* genes, suggesting that these isolates may have a more diverse reservoir and possibly a greater potential for type IV pili antigenic variation than invasive isolates. This difference could be linked to the expression of different classes of PilE, where class 1 consists of *pilE* gene and a *pilS* cassette adjacent in the genome. The class 1 PilE is present in many different CCs, and these isolates have more copies of *pilS* than class 2 pilE isolates [59]. The class 1 PilE isolates also express many different allelic variants of *pilE*. In isolates with class 2 PilE, the *pilE* and *pilS* are located in different regions of the genome and have fewer *pilS* copies [59]. The *pilE* genes that encode PilE class 2 have fewer allelic differences, compared to class 1 *pilE* isolates [59]. Class 2 *pilE* is almost exclusively found in a single clone (CC11), which makes it harder to confirm its association with invasiveness purely from the data gathered for this study. Although the association between the class 2 *pilE* and invasiveness was not strong enough to meet the significance threshold in the gene analysis, the high association scores, along with the significant association between invasiveness and several unique k-mers from class 2 *pilE*, provide some support for the role of class 2 *pilE* as a factor in invasiveness.

Many of the carriage-associated genetic variants identified in this study encode proteins expressed on the surface of *N. meningitidis *. Proteins on the surface of the bacteria are exposed to the immune system of the host, and this might make them prone to change the structure of these proteins to evade the immune responses. In *N. meningitidis *, this may be done through expressing different versions of a protein (antigenic variation) [72] or by switching between two phases of it (phase variation) [73]. During colonization, carriage may persist over a long period with the bacteria being exposed to the immune system for a longer time than during invasive disease [74]. The expression of particular surface structures or their variants may help colonizing isolates. Most of the genetic variants found here have previously been implicated as virulence or colonizing factors.

This study was limited by the few invasive isolates available due to the low incidence of IMD in Sweden. Also, almost half of these isolates belonged to serogroups W (CC11) and Y (CC23). Despite adjustment for population structure as part of the GWAS approach, some results in this study may have been affected by the lower frequency of genetic variants among the major serogroups of the invasive isolates in Sweden. Few GWAS studies have been performed on *N. meningitidis *, and few similarities were found to this study [23, 67, 75]. These studies have found carriage-derived k-mers in the *tspB* gene [67], associations of *tbpB* with invasive serogroup Y, cc23 isolates [75]*,* and another study found the gene NEIS0975 associated with invasive isolates [23], which we here found to be associated with carriage. These studies focused on one serogroup or CC, which may explain why we found different variants as we studied the entire *N. meningitidis* population from 2018 to 2019 in Sweden. Studies focusing on one serogroup or CC may be better at controlling population structure, but the variants that are identified may be linked to invasive isolates in the particular group studied and may not represent general traits across the population. One of these studies [67] was also based on a different GWAS approach, which also may have affected the results. A general limitation of GWAS approaches is their weak ability to distinguish spurious from real associations. This limitation was observed in this study, with several synonymous SNPs identified in close proximity to each other in the genome and sometimes in the same gene as non-synonymous SNPs. These SNPs do not impact directly on invasiveness; their spread into multiple branches of the phylogeny is likely caused by recombination events rather than regular evolutionary drift. For the k-mer analysis, multiple testing correction was performed based on the unique patterns of presence/absence observed rather than the total number of k-mers. This does increase the risk of false positives, but here it provides support for both the results of the SNP- and the gene-based analyses presented in this study, and for genes and genetic variants already believed to influence virulence in either *N. meningitidis* or *N. gonorrhoeae *. Since *N. meningitidis* undergoes frequent recombination, the genome is highly dynamic. This greatly increases the risk of identifying spurious associations between genotype and phenotype, as the introduction of a truly associated genotype into a new subpopulation via horizontal gene transfer will also include genomic traits that are not directly associated with a particular phenotype. The associations found in this study should therefore be evaluated critically and validated further. Some of the variants identified in this study will be included in further studies to support the present findings.

In conclusion, this study highlighted genetic variants associated with invasiveness and carriage in the *N. meningitidis* population in Sweden 2018–2019. These findings may be important in understanding the pathogenicity of *N. meningitidis* and identifying future vaccine targets. The associations found in this study need to be validated by additional GWAS studies and functional studies to understand whether, and how, these variants may impact invasiveness.

## Supplementary Data

Supplementary material 1
