# Supplementary material for: Genetic variants linked to the phenotypic outcome of invasive disease and carriage of Neisseria meningitidis
Source: Microb Genom. 2023 Oct 24;9(10):001124. doi: 10.1099/mgen.0.001124 (PMC10634450; doi:10.1099/mgen.0.001124)
Supplement: Supplementary material 1 [file mgen-9-1124-s001.pdf]

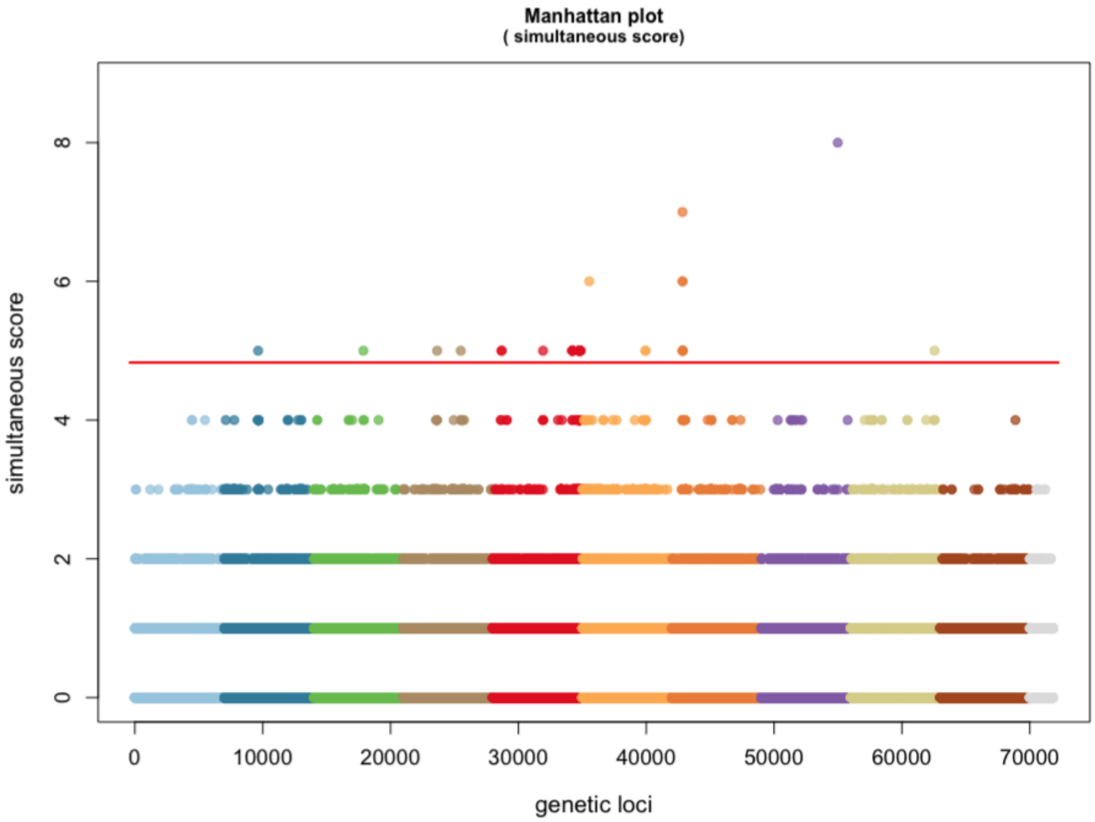


Fig. S1 Manhattan plot of Single nucleotide polymorphisms (SNPs) identified with the simultaneous score by treeWAS. The Manhattan plots displays the genetic locus on the x-axis and the score on the y-axis. The red line represents the significant threshold, and 31 significant SNPs were identified.

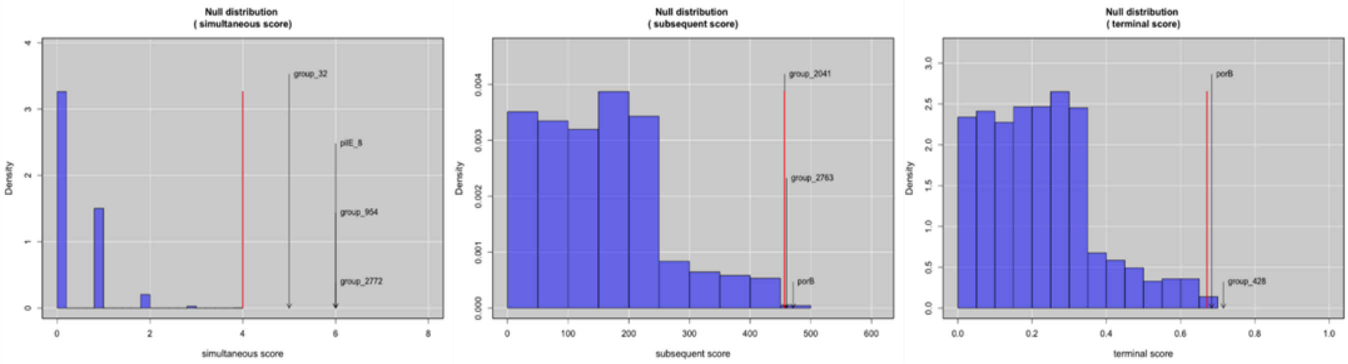


Fig. S2 Genes associated with invasive or carriage isolates identified with genome wide association study (GWAS). The distribution plots for the simultaneous, subsequent and the terminal scores are displayed. The red line represents a significant threshold, and significant genes were identified in each score

Table S1 Isolates included in the study

| PubMLST ID | Isolate ID | Serogroup | Sequence type (ST) | Clonal complex (CC) | Phenotype |
|---|---|---|---|---|---|
| 71448 | Mcbar-1541 | A | 4 | ST-4 complex | Carriage |
| 61290 | 18-114 | B | 34 | ST-32 complex | Invasive |
| 61298 | 18-174 | B | 803 | ST-32 complex | Invasive |
| 61309 | 18-299 | B | 269 | ST-269 complex | Invasive |
| 84110 | 18-352 | B | 6981 | ST-41/44 complex | Invasive |
| 84126 | 18-616 | B | 32 | ST-32 complex | Invasive |
| 70979 | 19-1136 | B | 32 | ST-32 complex | Invasive |
| 88978 | 19-212 | B | 2314 | ST-41/44 complex | Invasive |
| 88979 | 19-216 | B | 213 | ST-213 complex | Invasive |
| 85270 | 19-30 | B | 44 | ST-41/44 complex | Invasive |
| 88983 | 19-307 | B | 461 | ST-461 complex | Invasive |
| 91870 | 19-361 | B | 32 | ST-32 complex | Invasive |
| 91874 | 19-462 | B | 33 | ST-32 complex | Invasive |
| 91883 | 19-708-1 | B | 35 | ST-35 complex | Invasive |
| 70777 | 19-853 | B | 32 | ST-32 complex | Invasive |
| 93323 | Mcbar-1011 | B | 7460 | ST-32 complex | Carriage |
| 83785 | Mcbar-1029 | B | 32 | ST-32 complex | Carriage |
| 83786 | Mcbar-1032 | B | 7460 | ST-32 complex | Carriage |
| 83715 | Mcbar-104 | B | 213 | ST-213 complex | Carriage |
| 94476 | Mcbar-1167-4 | B | 10922 | ST-269 complex | Carriage |
| 83718 | Mcbar-119 | B | 809 | ST-35 complex | Carriage |
| 84604 | Mcbar-1304 | B | 32 | ST-32 complex | Carriage |
| 84607 | Mcbar-1314 | B | 948 | | Carriage |
| 84608 | Mcbar-1322 | B | 35 | ST-35 complex | Carriage |
| 84610 | Mcbar-1351 | B | 44 | ST-41/44 complex | Carriage |
| 71446 | Mcbar-1521 | B | 9812 | ST-213 complex | Carriage |
| 71447 | Mcbar-1538 | B | | | Carriage |
| 83710 | Mcbar-16 | B | 7460 | ST-32 complex | Carriage |
| 93261 | Mcbar-1660 | B | 809 | ST-35 complex | Carriage |
| 93265 | Mcbar-1710 | B | 7460 | ST-32 complex | Carriage |
| 93309 | Mcbar-1795 | B | 32 | ST-32 complex | Carriage |
| 93312 | Mcbar-1820 | B | 44 | ST-41/44 complex | Carriage |
| 93317 | Mcbar-1959 | B | 213 | ST-213 complex | Carriage |
| 93273 | Mcbar-2024 | B | 3469 | ST-4821 complex | Carriage |
| 93274 | Mcbar-2025 | B | 6058 | ST-41/44 complex | Carriage |
| 93281 | Mcbar-2113 | B | 12599 | | Carriage |
| 93285 | Mcbar-2172 | B | 213 | ST-213 complex | Carriage |
| 93289 | Mcbar-2415 | B | 7460 | ST-32 complex | Carriage |
| 83727 | Mcbar-244 | B | 32 | ST-32 complex | Carriage |
| 93245 | Mcbar-2475 | B | 7460 | ST-32 complex | Carriage |
| 93246 | Mcbar-2490 | B | 32 | ST-32 complex | Carriage |
| 93248 | Mcbar-2514 | B | 213 | ST-213 complex | Carriage |
| 94484 | Mcbar-2579 | B | 11836 | ST-461 complex | Carriage |
| 94487 | Mcbar-2619 | B | 34 | ST-32 complex | Carriage |

| | | | | | |
|---|---|---|---|---|---|
| 94489 | Mcbar-2635 | B | 213 | ST-213 complex | Carriage |
| 94490 | Mcbar-2647 | B | 7460 | ST-32 complex | Carriage |
| 94494 | Mcbar-2723 | B | 7460 | ST-32 complex | Carriage |
| 94497 | Mcbar-2739 | B | 11580 | ST-1157 complex | Carriage |
| 94498 | Mcbar-2752 | B | | | Carriage |
| 94504 | Mcbar-2813 | B | 32 | ST-32 complex | Carriage |
| 94505 | Mcbar-2817 | B | 7460 | ST-32 complex | Carriage |
| 94512 | Mcbar-3006 | B | 897 | | Carriage |
| 94513 | Mcbar-3055 | B | 1157 | ST-1157 complex | Carriage |
| 83732 | Mcbar-367 | B | 3327 | ST-865 complex | Carriage |
| 83743 | Mcbar-459 | B | 7460 | ST-32 complex | Carriage |
| 83745 | Mcbar-466 | B | 44 | ST-41/44 complex | Carriage |
| 83748 | Mcbar-489 | B | 213 | ST-213 complex | Carriage |
| 83755 | Mcbar-549 | B | 44 | ST-41/44 complex | Carriage |
| 94474 | Mcbar-589-4 | B | 213 | ST-213 complex | Carriage |
| 83760 | Mcbar-621 | B | 32 | ST-32 complex | Carriage |
| 83761 | Mcbar-622 | B | 32 | ST-32 complex | Carriage |
| 93292 | Mcbar-633 | B | 2003 | | Carriage |
| 83763 | Mcbar-640 | B | 32 | ST-32 complex | Carriage |
| 83765 | Mcbar-644 | B | 213 | ST-213 complex | Carriage |
| 83768 | Mcbar-664 | B | 32 | ST-32 complex | Carriage |
| 83769 | Mcbar-671 | B | 409 | ST-41/44 complex | Carriage |
| 83771 | Mcbar-697 | B | 8823 | | Carriage |
| 83772 | Mcbar-735 | B | 3469 | ST-4821 complex | Carriage |
| 83774 | Mcbar-791 | B | 809 | ST-35 complex | Carriage |
| 83713 | Mcbar-82 | B | 170 | ST-41/44 complex | Carriage |
| 83777 | Mcbar-867 | B | 213 | ST-213 complex | Carriage |
| 83781 | Mcbar-934 | B | 809 | ST-35 complex | Carriage |
| 83723 | Mcbar-175 | B | 897 | | Carriage |
| 61286 | 18-14 | C | 11 | ST-11 complex | Invasive |
| 61291 | 18-118 | C | 11 | ST-11 complex | Invasive |
| 61293 | 18-126 | C | 11 | ST-11 complex | Invasive |
| 61304 | 18-256 | C | 11 | ST-11 complex | Invasive |
| 61287 | 18-26 | C | 11 | ST-11 complex | Invasive |
| 61307 | 18-293 | C | 11 | ST-11 complex | Invasive |
| 84120 | 18-541 | C | 32 | ST-32 complex | Invasive |
| 85267 | 19-4 | C | 11 | ST-11 complex | Invasive |
| 91872 | 19-419 | C | 32 | ST-32 complex | Invasive |
| 91886 | 19-726 | C | 11 | ST-11 complex | Invasive |
| 70783 | 19-985 | C | 11 | ST-11 complex | Invasive |
| 70781 | 19-977 | C | 32 | ST-32 complex | Invasive |
| 83797 | Mcbar-1183 | C | 43 | ST-41/44 complex | Carriage |
| 93313 | Mcbar-1859 | C | 32 | ST-32 complex | Carriage |
| 93300 | Mcbar-2341 | C | 32 | ST-32 complex | Carriage |
| 83728 | Mcbar-249 | C | 32 | ST-32 complex | Carriage |
| 94492 | Mcbar-2685 | C | 213 | ST-213 complex | Carriage |
| 71449 | Mcbar-1586 | C | 467 | ST-269 complex | Carriage |

| | | | | | |
|---|---|---|---|---|---|
| 93311 | Mcbar-1819 | C | 14884 | | Carriage |
| 84129 | 18-641 | cnl | 41 | ST-41/44 complex | Invasive |
| 93250 | Mcbar-1011-3 | cnl | 7129 | | Carriage |
| 83783 | Mcbar-1021 | cnl | 53 | ST-53 complex | Carriage |
| 83789 | Mcbar-1084 | cnl | 823 | ST-198 complex | Carriage |
| 83790 | Mcbar-1094 | cnl | 823 | ST-198 complex | Carriage |
| 83717 | Mcbar-113 | cnl | 1136 | ST-1136 complex | Carriage |
| 83793 | Mcbar-1155 | cnl | 53 | ST-53 complex | Carriage |
| 83798 | Mcbar-1201 | cnl | 823 | ST-198 complex | Carriage |
| 83799 | Mcbar-1212 | cnl | 823 | ST-198 complex | Carriage |
| 83719 | Mcbar-122 | cnl | 823 | ST-198 complex | Carriage |
| 83800 | Mcbar-1226 | cnl | 41 | ST-41/44 complex | Carriage |
| 83720 | Mcbar-130 | cnl | 53 | ST-53 complex | Carriage |
| 84603 | Mcbar-1301 | cnl | 1649 | ST-1157 complex | Carriage |
| 83721 | Mcbar-131 | cnl | 53 | ST-53 complex | Carriage |
| 84606 | Mcbar-1310 | cnl | 53 | ST-53 complex | Carriage |
| 71445 | Mcbar-1510-2 | cnl | | | Carriage |
| 93257 | Mcbar-1623 | cnl | 198 | ST-198 complex | Carriage |
| 93259 | Mcbar-1634 | cnl | 823 | ST-198 complex | Carriage |
| 93260 | Mcbar-1640 | cnl | 823 | ST-198 complex | Carriage |
| 93263 | Mcbar-1701 | cnl | 845 | | Carriage |
| 93266 | Mcbar-1713 | cnl | 2384 | ST-198 complex | Carriage |
| 93267 | Mcbar-1739 | cnl | 823 | ST-198 complex | Carriage |
| 93269 | Mcbar-1752 | cnl | 198 | ST-198 complex | Carriage |
| 93270 | Mcbar-1762 | cnl | 823 | ST-198 complex | Carriage |
| 83724 | Mcbar-178 | cnl | 823 | ST-198 complex | Carriage |
| 93314 | Mcbar-1862 | cnl | 823 | ST-198 complex | Carriage |
| 93318 | Mcbar-1978 | cnl | 823 | ST-198 complex | Carriage |
| 83725 | Mcbar-200 | cnl | 1117 | ST-1117 complex | Carriage |
| 93319 | Mcbar-2008 | cnl | 823 | ST-198 complex | Carriage |
| 93272 | Mcbar-2009 | cnl | 3013 | | Carriage |
| 93277 | Mcbar-2037 | cnl | 823 | ST-198 complex | Carriage |
| 93278 | Mcbar-2045 | cnl | 2080 | ST-41/44 complex | Carriage |
| 93279 | Mcbar-2072 | cnl | 198 | ST-198 complex | Carriage |
| 93280 | Mcbar-2089 | cnl | 823 | ST-198 complex | Carriage |
| 93283 | Mcbar-2131 | cnl | 1136 | ST-1136 complex | Carriage |
| 93294 | Mcbar-2215 | cnl | | | Carriage |
| 93295 | Mcbar-2277 | cnl | 823 | ST-198 complex | Carriage |
| 93296 | Mcbar-2293 | cnl | 823 | ST-198 complex | Carriage |
| 93298 | Mcbar-2332 | cnl | 823 | ST-198 complex | Carriage |
| 93301 | Mcbar-2342 | cnl | 823 | ST-198 complex | Carriage |
| 93302 | Mcbar-2359 | cnl | 7129 | | Carriage |
| 93305 | Mcbar-2388 | cnl | 198 | ST-198 complex | Carriage |
| 93299 | Mcbar-2339 | cnl | 823 | ST-198 complex | Carriage |
| 93290 | Mcbar-2419 | cnl | 14883 | ST-1157 complex | Carriage |
| 93247 | Mcbar-2508 | cnl | 198 | ST-198 complex | Carriage |
| 93249 | Mcbar-2549 | cnl | 823 | ST-198 complex | Carriage |

| | | | | | |
|---|---|---|---|---|---|
| 94485 | Mcbar-2607 | cnl | 823 | ST-198 complex | Carriage |
| 94486 | Mcbar-2610 | cnl | 823 | ST-198 complex | Carriage |
| 94488 | Mcbar-2626 | cnl | 823 | ST-198 complex | Carriage |
| 94491 | Mcbar-2651 | cnl | 823 | ST-198 complex | Carriage |
| 94495 | Mcbar-2724 | cnl | 823 | ST-198 complex | Carriage |
| 83729 | Mcbar-276 | cnl | 53 | ST-53 complex | Carriage |
| 94499 | Mcbar-2767 | cnl | 7129 | | Carriage |
| 94501 | Mcbar-2790 | cnl | 823 | ST-198 complex | Carriage |
| 83730 | Mcbar-280 | cnl | 823 | ST-198 complex | Carriage |
| 94502 | Mcbar-2806 | cnl | 53 | ST-53 complex | Carriage |
| 94503 | Mcbar-2810 | cnl | 823 | ST-198 complex | Carriage |
| 94507 | Mcbar-2835 | cnl | | | Carriage |
| 94508 | Mcbar-2894 | cnl | 53 | ST-53 complex | Carriage |
| 94509 | Mcbar-2928 | cnl | 1649 | ST-1157 complex | Carriage |
| 94510 | Mcbar-2966 | cnl | 1649 | ST-1157 complex | Carriage |
| 94514 | Mcbar-3056 | cnl | 7129 | | Carriage |
| 93322 | Mcbar-333 | cnl | 7129 | | Carriage |
| 83734 | Mcbar-390 | cnl | 1136 | ST-1136 complex | Carriage |
| 83738 | Mcbar-419 | cnl | 198 | ST-198 complex | Carriage |
| 83739 | Mcbar-421 | cnl | 823 | ST-198 complex | Carriage |
| 83740 | Mcbar-427 | cnl | 3013 | | Carriage |
| 83741 | Mcbar-456 | cnl | 823 | ST-198 complex | Carriage |
| 83742 | Mcbar-457 | cnl | 53 | ST-53 complex | Carriage |
| 83747 | Mcbar-476 | cnl | 53 | ST-53 complex | Carriage |
| 83749 | Mcbar-490 | cnl | 3013 | | Carriage |
| 83751 | Mcbar-514 | cnl | 2080 | ST-41/44 complex | Carriage |
| 83752 | Mcbar-516 | cnl | 823 | ST-198 complex | Carriage |
| 83753 | Mcbar-531 | cnl | 823 | ST-198 complex | Carriage |
| 83756 | Mcbar-551 | cnl | 53 | ST-53 complex | Carriage |
| 83757 | Mcbar-554 | cnl | 823 | ST-198 complex | Carriage |
| 83712 | Mcbar-61 | cnl | 823 | ST-198 complex | Carriage |
| 83767 | Mcbar-663 | cnl | 3013 | | Carriage |
| 83770 | Mcbar-674 | cnl | 823 | ST-198 complex | Carriage |
| 83775 | Mcbar-831 | cnl | 823 | ST-198 complex | Carriage |
| 83779 | Mcbar-894 | cnl | 823 | ST-198 complex | Carriage |
| 83714 | Mcbar-91 | cnl | 1136 | ST-1136 complex | Carriage |
| 93251 | Mcbar-1028 | E | 254 | ST-254 complex | Carriage |
| 84601 | Mcbar-1297 | E | 60 | ST-60 complex | Carriage |
| 94496 | Mcbar-2725 | E | 1157 | ST-1157 complex | Carriage |
| 83737 | Mcbar-407 | E | 14023 | ST-1157 complex | Carriage |
| 83778 | Mcbar-884 | E | 14302 | | Carriage |
| 83780 | Mcbar-899 | E | 14023 | ST-1157 complex | Carriage |
| 83788 | Mcbar-1081 | E | 1157 | ST-1157 complex | Carriage |
| 71444 | Mcbar-1463-2 | E | 178 | ST-178 complex | Carriage |
| 93262 | Mcbar-1677 | E | 1157 | ST-1157 complex | Carriage |
| 83733 | Mcbar-369 | E | 178 | ST-178 complex | Carriage |
| 83746 | Mcbar-474 | E | 178 | ST-178 complex | Carriage |

| | | | | | |
|---|---|---|---|---|---|
| 83758 | Mcbar-586 | E | 1157 | ST-1157 complex | Carriage |
| 83764 | Mcbar-643 | E | 14022 | ST-1157 complex | Carriage |
| 94483 | Mcbar-2468 | ND | 11293 | ST-23 complex | Carriage |
| 61305 | 18-259 | NG | 23 | ST-23 complex | Invasive |
| 83795 | Mcbar-1169 | NG | 1649 | ST-1157 complex | Carriage |
| 83796 | Mcbar-1170 | NG | 1157 | ST-1157 complex | Carriage |
| 84602 | Mcbar-1299 | NG | 409 | ST-41/44 complex | Carriage |
| 84609 | Mcbar-1336 | NG | 1649 | ST-1157 complex | Carriage |
| 93256 | Mcbar-1529-3 | NG | 14882 | ST-41/44 complex | Carriage |
| 93304 | Mcbar-2371 | NG | 23 | ST-23 complex | Carriage |
| 93306 | Mcbar-2401 | NG | 1649 | ST-1157 complex | Carriage |
| 94493 | Mcbar-2699 | NG | 175 | ST-175 complex | Carriage |
| 94500 | Mcbar-2778 | NG | 1649 | ST-1157 complex | Carriage |
| 83736 | Mcbar-406 | NG | 1649 | ST-1157 complex | Carriage |
| 83782 | Mcbar-984 | NG | 1649 | ST-1157 complex | Carriage |
| 83750 | Mcbar-513 | NG | 23 | ST-23 complex | Carriage |
| 61294 | 18-151 | W | 11 | ST-11 complex | Invasive |
| 61292 | 18-122 | W | 11 | ST-11 complex | Invasive |
| 61296 | 18-164 | W | 11 | ST-11 complex | Invasive |
| 61297 | 18-166 | W | 11 | ST-11 complex | Invasive |
| 61299 | 18-185 | W | 11 | ST-11 complex | Invasive |
| 61300 | 18-192 | W | 11 | ST-11 complex | Invasive |
| 61306 | 18-271 | W | 11 | ST-11 complex | Invasive |
| 84112 | 18-378 | W | 11 | ST-11 complex | Invasive |
| 84114 | 18-391 | W | 11 | ST-11 complex | Invasive |
| 84116 | 18-402 | W | 11 | ST-11 complex | Invasive |
| 84118 | 18-469 | W | 11 | ST-11 complex | Invasive |
| 84119 | 18-490 | W | 11 | ST-11 complex | Invasive |
| 83924 | 18-520 | W | 11 | ST-11 complex | Invasive |
| 84121 | 18-554 | W | 11 | ST-11 complex | Invasive |
| 84123 | 18-567 | W | 11 | ST-11 complex | Invasive |
| 61288 | 18-62 | W | 11 | ST-11 complex | Invasive |
| 84127 | 18-623 | W | 11 | ST-11 complex | Invasive |
| 85262 | 18-671 | W | 11 | ST-11 complex | Invasive |
| 85263 | 18-734 | W | 11 | ST-11 complex | Invasive |
| 85264 | 18-742 | W | 11 | ST-11 complex | Invasive |
| 61289 | 18-87 | W | 11 | ST-11 complex | Invasive |
| 70785 | 19-1057 | W | 11 | ST-11 complex | Invasive |
| 70786 | 19-1110 | W | 1281 | ST-22 complex | Invasive |
| 71427 | 19-1173 | W | 11 | ST-11 complex | Invasive |
| 71921 | 19-1182 | W | 11 | ST-11 complex | Invasive |
| 71922 | 19-1196 | W | 11 | ST-11 complex | Invasive |
| 88980 | 19-219 | W | 14492 | ST-11 complex | Invasive |
| 88981 | 19-228 | W | 11 | ST-11 complex | Invasive |
| 85269 | 19-29 | W | 11 | ST-11 complex | Invasive |
| 88984 | 19-328 | W | 11 | ST-11 complex | Invasive |
| 91871 | 19-397 | W | 11 | ST-11 complex | Invasive |

| | | | | | |
|---|---|---|---|---|---|
| 85271 | 19-50 | W | 11 | ST-11 complex | Invasive |
| 85272 | 19-59 | W | 11 | ST-11 complex | Invasive |
| 70774 | 19-548 | W | 11 | ST-11 complex | Invasive |
| 91876 | 19-508 | W | 11 | ST-11 complex | Invasive |
| 91879 | 19-559 | W | 11 | ST-11 complex | Invasive |
| 91882 | 19-656 | W | 11 | ST-11 complex | Invasive |
| 91885 | 19-719 | W | 11 | ST-11 complex | Invasive |
| 91888 | 19-732 | W | 11 | ST-11 complex | Invasive |
| 70775 | 19-756 | W | 11 | ST-11 complex | Invasive |
| 70776 | 19-806 | W | 11 | ST-11 complex | Invasive |
| 88974 | 19-87 | W | 11 | ST-11 complex | Invasive |
| 70778 | 19-909 | W | 11 | ST-11 complex | Invasive |
| 84612 | Mcbar-1399 | W | 11 | ST-11 complex | Carriage |
| 83766 | Mcbar-646 | X | 3151 | ST-1157 complex | Carriage |
| 61295 | 18-161 | Y | 23 | ST-23 complex | Invasive |
| 61301 | 18-208 | Y | 23 | ST-23 complex | Invasive |
| 61302 | 18-210 | Y | 23 | ST-23 complex | Invasive |
| 61303 | 18-215 | Y | 23 | ST-23 complex | Invasive |
| 61308 | 18-296 | Y | 1655 | ST-23 complex | Invasive |
| 84111 | 18-375 | Y | 23 | ST-23 complex | Invasive |
| 84115 | 18-400 | Y | 1655 | ST-23 complex | Invasive |
| 84117 | 18-411 | Y | 23 | ST-23 complex | Invasive |
| 84124 | 18-573 | Y | 23 | ST-23 complex | Invasive |
| 84125 | 18-607 | Y | 23 | ST-23 complex | Invasive |
| 84128 | 18-637 | Y | 23 | ST-23 complex | Invasive |
| 85265 | 18-752 | Y | 3587 | ST-23 complex | Invasive |
| 84122 | 18-563 | Y | 23 | ST-23 complex | Invasive |
| 91877 | 19-509 | Y | 23 | ST-23 complex | Invasive |
| 70787 | 19-1111 | Y | 23 | ST-23 complex | Invasive |
| 85266 | 19-3 | Y | 23 | ST-23 complex | Invasive |
| 88975 | 19-160 | Y | 13126 | ST-167 complex | Invasive |
| 85268 | 19-18 | Y | 23 | ST-23 complex | Invasive |
| 88976 | 19-187 | Y | 23 | ST-23 complex | Invasive |
| 88977 | 19-192 | Y | 23 | ST-23 complex | Invasive |
| 88982 | 19-241 | Y | 23 | ST-23 complex | Invasive |
| 91873 | 19-424 | Y | 23 | ST-23 complex | Invasive |
| 91875 | 19-463 | Y | 23 | ST-23 complex | Invasive |
| 91878 | 19-511 | Y | 1655 | ST-23 complex | Invasive |
| 91880 | 19-611 | Y | 23 | ST-23 complex | Invasive |
| 91881 | 19-616 | Y | 23 | ST-23 complex | Invasive |
| 91887 | 19-727 | Y | 23 | ST-23 complex | Invasive |
| 85273 | 19-84 | Y | 1655 | ST-23 complex | Invasive |
| 70779 | 19-926 | Y | 23 | ST-23 complex | Invasive |
| 70780 | 19-963 | Y | 23 | ST-23 complex | Invasive |
| 70782 | 19-978 | Y | 23 | ST-23 complex | Invasive |
| 70784 | 19-999 | Y | 23 | ST-23 complex | Invasive |
| 83792 | Mcbar-1105 | Y | 11293 | ST-23 complex | Carriage |

| | | | | | |
|---|---|---|---|---|---|
| 83794 | Mcbar-1160 | Y | 11293 | ST-23 complex | Carriage |
| 83801 | Mcbar-1234 | Y | 23 | ST-23 complex | Carriage |
| 84605 | Mcbar-1307 | Y | 11293 | ST-23 complex | Carriage |
| 84611 | Mcbar-1356 | Y | 1655 | ST-23 complex | Carriage |
| 83722 | Mcbar-158 | Y | 23 | ST-23 complex | Carriage |
| 93258 | Mcbar-1632 | Y | 1655 | ST-23 complex | Carriage |
| 93310 | Mcbar-1807 | Y | 1655 | ST-23 complex | Carriage |
| 93315 | Mcbar-1884 | Y | 913 | ST-60 complex | Carriage |
| 93316 | Mcbar-1918 | Y | 11293 | ST-23 complex | Carriage |
| 93321 | Mcbar-2015 | Y | 11293 | ST-23 complex | Carriage |
| 93275 | Mcbar-2035-3 | Y | 11293 | ST-23 complex | Carriage |
| 93276 | Mcbar-2036 | Y | 11293 | ST-23 complex | Carriage |
| 83711 | Mcbar-21 | Y | 23 | ST-23 complex | Carriage |
| 93287 | Mcbar-2266 | Y | 11293 | ST-23 complex | Carriage |
| 93297 | Mcbar-2296 | Y | 23 | ST-23 complex | Carriage |
| 93288 | Mcbar-234 | Y | 23 | ST-23 complex | Carriage |
| 93244 | Mcbar-2473 | Y | 11293 | ST-23 complex | Carriage |
| 94506 | Mcbar-2834 | Y | 913 | ST-60 complex | Carriage |
| 83735 | Mcbar-396 | Y | 1624 | ST-167 complex | Carriage |
| 83744 | Mcbar-462 | Y | 11293 | ST-23 complex | Carriage |
| 83754 | Mcbar-536 | Y | 1655 | ST-23 complex | Carriage |
| 83759 | Mcbar-618 | Y | 9083 | ST-23 complex | Carriage |
| 83762 | Mcbar-634 | Y | 23 | ST-23 complex | Carriage |
| 83776 | Mcbar-865 | Y | 23 | ST-23 complex | Carriage |
| 93268 | Mcbar-1743 | Y | 23 | ST-23 complex | Carriage |
| 93320 | Mcbar-2011 | Y | 23 | ST-23 complex | Carriage |
| 93284 | Mcbar-2171 | Y | 23 | ST-23 complex | Carriage |
| 71443 | Mcbar-1425-2 | Y | 23 | ST-23 complex | Carriage |
| 93286 | Mcbar-2216 | Y | 23 | ST-23 complex | Carriage |
| 93254 | Mcbar-1467-3 | Y | 23 | ST-23 complex | Carriage |
| 93303 | Mcbar-2366 | Y | 23 | ST-23 complex | Carriage |
| 94511 | Mcbar-2980 | Y | 23 | ST-23 complex | Carriage |
| 83787 | Mcbar-1035 | Y | 1649 | ST-1157 complex | Carriage |
| 83802 | Mcbar-1244 | Y | 4963 | ST-103 complex | Carriage |
| 83716 | Mcbar-108 | Z | 3882 | | Carriage |
| 71439 | Mcbar-125-2 | Z | 5953 | | Carriage |
| 83773 | Mcbar-778 | Z | 3882 | | Carriage |

Table S2 Open reading frames (ORF) of the Meningococcal disease associated (MDA) phage and the corresponding NEIS genes.

| MDA phage ORF | NCBI (isolate Z2491) | NEIS genes (PubMLST) | Product (PubMLST) |
|---|---|---|---|
| ORF1 | NMA1792 | NEIS0031<br>NEIS0277<br>NEIS1861 | Phage replication initiation factor |
| ORF2 | NMA1793 | NEIS0030<br>NEIS1862<br>NEIS2452<br>NEIS2460 | Hypothetical protein |
| ORF3 | NMA1794 | NEIS0029<br>NEIS1863<br>NEIS2453<br>NEIS2459 | Hypothetical protein |
| ORF4 | NMA1795 | NEIS0028<br>NEIS1864 | Hypothetical protein |
| ORF5 | NMA1796 | NEIS0027<br>NEIS1865 | Hypothetical protein |
| ORF6 | NMA1797 | NEIS0025<br>NEIS1715<br>NEIS1866 | TspB protein |
| ORF7 | NMA1798 | NEIS0024<br>NEIS1867 | Hypothetical protein |
| ORF8 | NMA1799 | NEIS0023<br>NEIS1868 | Putative zonular occludens toxin-like protein/ hypothetical protein |
